# Factors Associated With Alzheimer’s Disease Patients’ Caregiving Status and Family Caregiving Burden in China

**DOI:** 10.3389/fnagi.2022.865933

**Published:** 2022-03-16

**Authors:** Yuxian Li, Fangda Leng, Qi Xiong, Jiong Zhou, Ailian Du, Feiqi Zhu, Xiaowen Kou, Wei Sun, Luzeng Chen, Huali Wang, Hengge Xie, Feng Gao, Haiqiang Jin, Yongan Sun

**Affiliations:** ^1^Department of Neurology, Peking University First Hospital, Peking University, Beijing, China; ^2^Department of Neurology, Beijing Tiantan Hospital, Capital Medical University, Beijing, China; ^3^Health Service Department of the Guard Bureau of the Joint Staff Department, Beijing, China; ^4^Department of Neurology, The Second Affiliated Hospital, School of Medicine, Zhejiang University, Zhejiang, China; ^5^Department of Neurology, Shanghai Tongren Hospital, Shanghai Jiao Tong University School of Medicine, Shanghai, China; ^6^Department of Neurology, Shenzhen Luohu People’s Hospital, Shenzhen, China; ^7^Health Times, The People’s Daily, Beijing, China; ^8^Department of Ultrasonography, Peking University First Hospital, Peking University, Beijing, China; ^9^Department of Psychiatry, Peking University Sixth Hospital, Peking University, Beijing, China; ^10^Department of Neurology, Second Medical Center of Chinese PLA General Hospital, Beijing, China

**Keywords:** Alzheimer’s disease, home care, caregiver burden, institutional care, care preference, caregiving

## Abstract

**Background:**

The increasing prevalence of Alzheimer’s disease (AD) has emerged as a major challenge worldwide. China as the most populous country in the globe is amid rapid aging of its population, highlighting the need for appropriate social and medical policies to meet the challenge. The current multicenter cross-sectional observational study aims to provide understanding of the current status of caring given to AD patients in China and investigate the factors that influence the family burden as well as the choice of care given to AD patients.

**Methods:**

A total of 1,675 patients with probable AD from 30 provincial regions of mainland China were enrolled in the current study from August 2019 to December 2019. We analyzed the caregiving status and its relationship with family burden and various socio-economical and medical factors.

**Results:**

In the current study, 90.87% of the AD patients enrolled adopted family care. The choice of caregiving method was influenced by factors including age (>80 years old, OR 0.648; 95% CI, 0.427–0.983), overall family burden (high, OR 0.574; 95% CI, 0.0.373–0.884), patients’ income (OR 0.511; 95% CI, 0.330–0.789) and self-care ability (OR 0.329; 95% CI, 0.183–0.588).

**Conclusion:**

Family care is the primary method of care for AD patients in China and the institutional care system for AD patients is still underprepared in China.

## Introduction

China as the world’s most populous country is amid the rapid aging of its population, which is accompanied by a drastic increase of dementia prevalence (Pei et al., 2014). Alzheimer’s disease (AD) is the most common type of senile dementia, accounting for over 60% of all-cause dementia (Alzheimer’s Association, 2018). It has been estimated that the prevalence of AD among senior citizens above 65 year old is 3.21% and increases substantially with age among Chinese population (Jia et al., 2014), which has a considerable sociological and economic impact on Mainland China. It is estimated that the average socioeconomic cost of AD per patient per year is 19,144 USD in mainland China (Jia et al., 2018). The annual total cost is predicted to reach 1.89 trillion USD in 2030 in China, rendering the care of AD patients beyond a medical issue, but also an outstanding sociological problem.

Among the issues that awaits to be addressed are how to provide AD patients with appropriate care and to reduce the burden for the families. While it has been suggested that family care might be a better method of care for patients with AD considering its emotional and psychological comfort (Luppa et al., 2010), studies from high-income countries have demonstrated that institutional care offers better functional outcome compared to family care (Afram et al., 2014; Lee et al., 2019). Further, the demands of caring responsibilities change with the stages of disease, which can be a great challenge for the family. In Europe, it has been reported that the emotional and psychological distress to family caregivers enforced by Alzheimer’s disease urged families to seek professional care for the patients (Bokberg et al., 2015). However, the domestic situation with regard to care giving for AD patients remains to be investigated in China, as such socio-economical issues are highly entangled by cultural and economic factors, with huge variabilities among different nations.

As an example, in western society, the choice of home or institutional care of a patient may mainly depend on personal needs, financial situations, and the accessibility of professional care (Genet et al., 2011), while in China, sending elderlies to care homes may be seen as a betrayal to the family. To date, few studies have been conducted on the caregiving status and the relationship between caregiver burden and patient factors in China. The lack of understanding on the current status of care given to domestic AD patients, together with the short of analytical data on the underlying factors have left policymakers in dark to improve welfare for AD patients and their families. In the current study, we hypothesized that the burden of family members is a major factor that influences the decision of home or institutional care for AD patients in China. We aimed to investigate the current caregiving status and burden as well as to analyze the relationship between caregiver burden and patient factors to suggest ideas for policy and research programs on chronic diseases.

## Materials and Methods

### Design and Samples

This study was approved by the Ethics Committee of Peking University First Hospital (PUFH-2019-141), the leading institution of the study, and local ethic committees in all participating centers. informed consent were obtained from all patients and family members.

**FIGURE 1:**
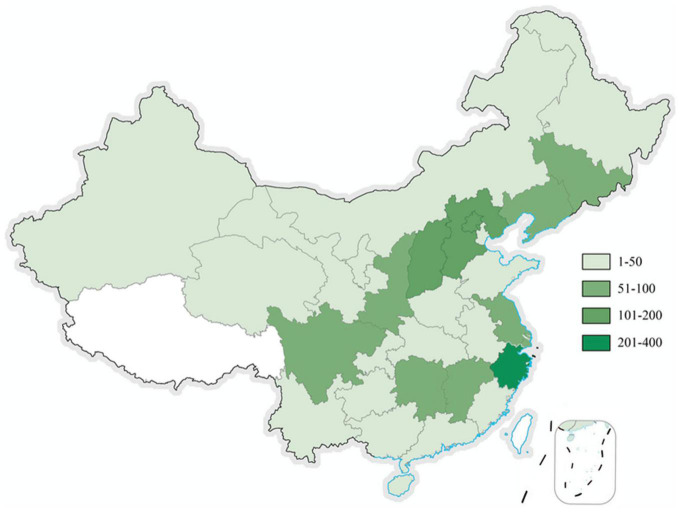
Geographical distribution of participants in the current study.

This is a large-sample, multi-center, cross-sectional study performed between August 2019 and December 2019. We collected data from 30 provincial, municipal, and autonomous regions of mainland China. Among them, the eastern provinces, such as Zhejiang, Beijing, and Hebei contributed the most participants (Figure 1).

The recruitment of participants was based on the hierarchical healthcare structure in China, where there are 3 levels of healthcare infrastructures, with the first level being community clinics and the third level being the local health centers. The participants were referred to the local health centers by local clinics or sought consultation directly from the health centers, where the patients were diagnosed as clinical probable AD by qualified neurology specialists from the local health centers. The diagnosis of probable AD dementia was based on the diagnostic criteria established by the National Institute of Aging and Alzheimer’s Association (NIA-AA) in 2011 (McKhann et al., 2011). Other inclusion criteria include: (1) capability to give written consent, (2) next of kin’s consent to participate, and (3) >5 years of education. Patients with other neurological diseases that are associated with cognitive impairment and major psychiatric disorders were excluded. In particular, patients with signs and symptoms suggesting other types of dementia, such as Parkinson’s disease dementia, Lewy body dementia, frontotemporal dementia, primary progressive aphasia, vascular dementia and mixed dementia.

All medical staff who conducted the study were given comprehensive training on the usage of the questionnaire used in the study. Family members and professional caregivers (for patients in nursing facilities) of the patients completed the questionnaire under the guidance of doctors. For those who had already been in care homes, the family members were instructed to answer the questionnaire according to the situation when the patients were last at home. The current study focused on the perceived care burden by family members as (1) in the vast majority of scenarios it were the family members who accompanied the patients to the health centers, and (2) the decisions of home care vs. institutional care were made by the patients and their families.

### Measures and Data Collection

We designed a comprehensive questionnaire, which includes demographic characteristics, household income, medical history with regard to AD, care situation, and burden to the family. The caregiving status of the patients was divided into two categories, including family care and institutional care. Family care was defined as situations in which family members, including spouses, children, grandchildren, and other relatives, take care of AD patients with or without the help of a health professional. Institutional care indicates that the patients are taken care of by professionals in nursing facilities. Similarly, self-care ability was divided into two classes: basic self-care and partially or completely dependent. The type of caregiving was assessed by caregivers based on whether the patients could take care of themselves in their daily lives.

The caregiver burden inventory (CBI) (Chinese version) was used to describe the multidimensional burden of the caregivers and to distinguish related factors of different burden dimensions (Novak and Guest, 1989). The translation and validation of CBI in Chinese population was performed by Chou et al. (2002). The inventory consists of 24 questions that refer to five dimensions: time-dependence burden (questions 1–5), developmental burden (questions 6–10), physical burden (questions 11–14), social burden (questions 15–18), and emotional burden (questions 19–24). Each item was graded on a 4-point Likert scale according to the degree of each situation. A high score represented a high burden and the total score is 96. The point range from 0 to 32 was considered as a low burden, from 33 to 64 as a medium burden, and from 65 to 96 as a high burden.

The questionnaires were examined manually to ensure completeness and effectiveness immediate after the interview by staff. Incomplete questionnaire and those with obvious contradictory answers to the interview were considered invalid.

### Statistical Analyses

Patients and family members’ characteristics, including gender, age, housing condition, education, and annual household income were presented using descriptive statistics. Quantitative variables were examined for normal distribution and presented as the mean ± standard deviation. Independent-samples *t*-tests and one-way ANOVA were performed for group-wise comparisons of continuous variables. Chi-square tests were performed to examine cross-group differences of categorical variables. To examine the reliability performance of CBI in the settings of current study, Cronbach’s alpha was calculated for all the items in the inventory and each of the five dimensions. Cronbach’s alpha efficient >0.9 was considered to indicate excellent internal consistency, and >0.8 was considered to indicate good consistency. Factors with *P* < 0.2 in group-wise comparisons were then entered to binary logistic regression models to evaluate the influence of each factors in the choice of caregiving for AD patients, controlling for age and gender. All data analyses were performed using SPSS 26.0 (SPSS Inc., Chicago, IL, United States). Differences were considered statistically significant when the *P*-value was less than 0.05.

## Results

### Patient Characteristics

A total of 1,694 people participated in the survey. After eliminating the erroneous and invalid questionnaires, 1,675 valid questionnaires were left, resulting in an effectiveness of 98.88%.

The patients characteristics are presented in Table 1. The 1675 AD patients were from 30 provincial, municipal, and autonomous regions of mainland China, including 650 (38.81%) men and 1,025 (61.19%) women. The participation of patients in urban areas was higher than that in rural areas (79.76 vs. 20.24%). The geographical distribution of the patients is illustrated in Figure 1.

**TABLE 1:** Characteristics of caregivers and patients with AD (*N* = 1675).

Variable	*N*	%
**Patients**		
**Sex**		
Male	650	38.81%
Female	1025	61.19%
**Age**		
<70	580	34.63%
71–80	546	32.60%
>80	549	32.78%
**Living area**		
Urban	1336	79.76%
Rural	339	20.24%
**Education level, years**		
5–6	492	29.37%
7–12	776	46.33%
>12	407	24.30%
**Self-care ability**		
Basic self-care	424	25.31%
Partially or wholly dependent	1251	74.69%
**Annual patient’s income (10,000yuan)**		
<3	767	45.79%
3–5	522	31.16%
>5	386	23.04%
**Annual household income (10,000yuan)**		
<5	611	36.48%
5–15	802	47.88%
>15	262	15.64%
**Caregivers and Treatment**		
**Caregiving status**		
Family care	1522	90.87%
Nursing facility	153	9.13%
**Dementia drug application**		
On medication	1278	75.30%
Used and withdrew	259	15.46%
Untreated	138	8.24%
**Annual medical cost (10,000 yuan)**		
<1	821	49.01%
1—2.4	585	34.93%
>2.4	269	16.06%
**Annual care cost (10,000 yuan)**		
<2	542	32.36%
2–6	573	34.21%
>6	266	15.88%
Unclear	294	17.55%
**Caregiver burden**		
Low	484	28.90%
Medium	955	57.01%
High	236	14.09%

### Comparison of Characteristics Between Home-Cared and Institution-Cared Patients

Of the 1,675 AD patients sampled, 1522 (90.87%) patient chose family care and 153 (9.13%) patients lived in care homes. To identify the factors associated with the choice of care, we analyzed the characteristics of the patients’ caregiving status (Table 2). Home cared and institution cared patients did not differ by gender or education level. Patients who were above 80 years (*P* = 0.003) and living in urban areas (*P* < 0.001) were more likely to choose institutional care compared to those who were younger and in rural regions. Concerning economic status, the choice of patient care is related to the annual income of the patient (*P* < 0.001). A greater proportion of patients living in nursing homes belong to a higher income group; however, no significant difference was found between annual household income groups (*P* = 0.122). According to self-care ability and CBI score, patients who are less capable of taking care of themselves and who impose a large burden to family caregivers tended to choose nursing facilities (*P* = 0.001).

**TABLE 2:** The caregiving status of the familial caregiver of patients with AD in relation to patient and caregiver characteristics (*N* = 1675).

	Family care *N* = 1522 (%)	Institutional care *N* = 153 (%)	*P*-value
Sex			0.948
Male	591 (38.83)	59 (38.56)	
Female	931 (61.17)	94 (61.44)	
Age			0.003
<70	538 (35.35)	42 (27.45)	
71–80	506 (33.25)	40 (26.14)	
>80	478 (31.41)	71 (46.41)	
Living area			<0.001
Urban	1194 (78.45)	142 (92.81)	
Rural	328 (21.55)	11 (7.19)	
Education level, years			0.052
5–6	457 (30.03)	35 (22.88)	
6–12	706 (46.39)	70 (45.75)	
>12	359 (23.59)	48 (31.37)	
Annual patient’s income (1,0000 yuan)			<0.001
<3	724 (47.57)	43 (28.10)	
>3	798 (52.43)	110 (71.90)	
Annual household income (1,0000 yuan)			0.122
<5	564 (37.06)	47 (30.72)	
5–15	728 (47.83)	74 (29.25)	
>15	230 (15.11)	32 (20.92)	
Self-care ability			<0.001
Basic self-care	408 (26.81)	16 (10.46)	
Partially or wholly dependent	1114 (73.19)	137 (89.54)	
Family care burden			0.001
Low	450 (29.57)	34 (22.22)	
Medium	872 (57.29)	83 (54.25)	
High	200 (13.14)	36 (23.53)	

### Regression Analysis of Factors Associated With Choice of Care

We used binary logistic regression analysis to identify the relevant factors for choosing nursing facilities for patients with AD (Table 3). Urban patients are more likely to choose nursing homes than rural patients (OR = 2.374; 95% CI, 1.228–4.588). Impaired self-care ability was also a predictor of choosing nursing homes over family care (OR = 0.329; 95% CI, 0.183–0.588). The family care burden is also a related factor, with families having higher perceived care burden being more inclined to seek institutional care (OR = 0.574; 95% CI,0.373–0.884).

**TABLE 3:** Logistic regression models for the caregiving status of AD patients (*N* = 1675).

	OR	(95% CI)		*P*-value
**Age**				
<70	Ref.			
71–80	0.744	0.489	1.131	0.167
>80	0.648	0.427	0.983	0.041
**Living area**				
Rural	Ref.			
Urban	2.374	1.228	4.588	0.010
**Annual patient’s income (10,000 yuan)**				
<3	Ref.			
>3	0.511	0.330	0.789	0.003
**Annual household income (10,000 yuan)**				
<5	Ref.			
5–15	0.877	0.512	1.500	0.631
>15	0.726	0.462	1.141	0.165
**Self-care ability**				
Basic self-care	Ref.			
Partially or wholly dependent	0.329	0.183	0.588	<0.001
**Family care burden**				
Low	Ref.			
Medium	0.713	0.411	1.235	0.227
High	0.574	0.373	0.884	0.012

### Internal Consistency of the Chinese Version Caregiver Burden Inventory Scale

In the current study, the overall Cronbach’s alpha of the CBI scale (24 items) was 0.950 (95% CI, 0.946–0.953); the time-dependence burden (5 items) dimension had a Cronbach’s alpha of 0.934 (95% CI, 0.929–0.940); developmental burden (five items) dimension had Cronbach’s alpha of 0.939 (95% CI, 0.933–0.944); physical burden (four items)’s Cronbach’s alpha was 0.954 (95% CI, 0.950–0.959); social burden (five items) dimension’s Cronbach’s alpha was 0.831 (95% CI, 0.0.815–0.845); and the Cronbach’s alpha of emotional burden (five items) was 0.854 (95% CI, 0.835–0.869). These coefficients indicated that the CBI scale had an excellent overall internal consistency and the sub-domains offered at least good reliability in the current study.

### Burden on Caregivers of Alzheimer’s Disease Patients

In this study, 25.31% of the patients could take basic care of themselves. The other 74.69% of patients were partially or wholly dependent on others for care. About 36.48% of patients were completely dependent on caregivers, indicating that a large proportion of AD patients had severe disabilities. According to the CBI scores, 28.90% of families had a relatively low level of burden, 14.09% of families had a high level of burden, and the remaining 57.01% had a medium level of burden. From the perspective of burden classification, all five dimensions of burden were separately calculated in the two different groups (Table 4). All aspects of family burden significantly increased when patients were not able to take care of themselves (*P* < 0.001). Caregiving status was also associated with burden grade. Families of patients who were at nursing facilities had a higher total burden of care (when the patients were last at home) compared to those home-cared (*P* < 0.001). In addition, in other aspects such as time-dependence, development limitation, health, and social contact, families bear a higher burden before the patients were sent to a nursing home than those home-cared.

**TABLE 4:** CBI scores of the family of patients with AD based on the caregiving status.

Burden classification	*n*	Time-dependence (−x ± s,score)	*P*-value	Developmental (−x ± s,score)	*P*-value	Physical (−x ± s,score)	*P*-value	Social (−x ± s,score)	*P*-value	Emotional (−x ± s,score)	*P*-value	Total (−x ± s,score)	*P*-value
Self-care ability			<0.001		<0.001		<0.001		<0.001		<0.001		<0.001
Basic self-care	424	6.53 ± 4.65		7.62 ± 5.62		4.56 ± 4.16		3.30 ± 3.36		3.02 ± 3.63		25.04 ± 18.02	
Partially or wholly dependent	1251	15.45 ± 4.20		14.09 ± 4.87		9.60 ± 4.42		5.90 ± 4.07		4.82 ± 4.65		49.87 ± 16.81	
Care-giving status			<0.001		0.001		<0.001		0.008		0.510		<0.001
Family care	1522	12.98 ± 5.81		12.30 ± 5.74		8.17 ± 4.83		5.16 ± 4.02		4.34 ± 4.41		42.95 ± 20.05	
Nursing facility	153	15.29 ± 5.34		13.96 ± 6.14		9.90 ± 5.07		6.14 ± 4.41		4.62 ± 5.17		49.92 ± 21.03	

The family burden was affected by both the self-care ability and caregiving status of the patients at the same time. To exclude confounding factors, we used stratified correlation analysis to determine the impact of self-care ability and caregiving status on the burden of caregivers (Table 5). There was no significant difference of family burden, either family care or nursing facility (*P* = 0.520), if the patients could take care of themselves. However, if the patients were incapable of self-care, the overall family burden was higher carer-dependent AD patients (*P* = 0.015), and the differences mainly manifested in the burden of time-dependence (P = 0.002) and the health of caregivers (*P* = 0.008).

**TABLE 5:** CBI scores of family members with different care status after classification according to patient self-care ability.

Self-care ability	*n*	Time-Dependence (−x ± s,score)	*P*-value	Developmental (−x ± s,score)	*P*-value	Physical (−x ± s,score)	*P*-value	Social (−x ± s,score)	*P*-value	Emotional (−x ± s,score)	*P*-value	Total (−x ± s,score)	*P*-value
Basic self-care			0.163		0.557		0.902		0.873		0.561		0.520
Family care	408	6.59 ± 4.69		7.65 ± 5.61		4.57 ± 4.14		3.30 ± 3.29		3.04 ± 3.64		25.15 ± 17.95	
Nursing facility	16	4.94 ± 3.21		6.81 ± 5.79		4.44 ± 4.84		3.50 ± 4.97		2.50 ± 3.63		22.19 ± 20.26	
Partially or wholly dependent			0.002		0.118		0.008		0.094		0.901		0.015
Family care	1114	15.32 ± 4.20		14.01 ± 4.76		9.48 ± 4.38		5.84 ± 4.05		4.81 ± 4.57		49.46 ± 16.53	
Nursing facility	137	16.50 ± 4.08		14.80 ± 5.63		10.54 ± 4.72		6.45 ± 4.26		4.87 ± 5.28		53.15 ± 18.66	

## Discussion

Alzheimer’s disease is a progressive neurodegenerative disease, which causes cognitive decline in multiple cognitive domains including language, visuospatial, executive function, complex attention, perceptual-motor, social cognition, and most commonly, memory (McKhann et al., 2011). Behavioral and psychological symptoms gradually occur in AD patients, resulting in disability and most patients are completely dependent at later stages of the disease (Atri, 2019).

In the current study, the factors associated with the choice of home or institutional care for AD patients in China. We found that age (>80 years), living in urban areas, higher patient annual income, inability of self-care and high family care burden were associated with higher probability of choosing institutional care for AD patients in China.

Our results suggest that family care is the most common choice for families of AD patients in China, which is a common phenomenon in developing countries especially in Asia (Prince and Dementia Research Group, 2004). While increasing numbers of nursing facilities is being set up, Chinese families with AD patients have a low preference for institutional care (Table 6). Aside from the subjective reasons (disapproval by family members or patients), our questionnaire suggested that the primary reasons against institutional care were economic burden (34.9%) and insufficient service provided by the institutions (34.5%). Many people view that nursing homes cannot provide individual care to patients. It is noteworthy that some family caregivers (4.4% of all home-cared patients and 32.6% of all patients who have experienced institutional care) mention that the patients who earlier chose to live in a nursing home, for various reasons, often decide to leave the nursing home and return to family care. The striking proportion of AD patients dropping from nursing homes indicates deficiencies of Chinese institutional care system’s infrastructure and proficiency, which is evidenced by a lack of care professionals’ knowledge on dementia (Wang Y. et al., 2018). The low subjective preference of Chinese family for institutional care might be related to traditional cultural heritage, as Chinese people attach great importance to filial piety. In fact, during interview, some patients living nursing homes admitted feeling abandoned by their families. However, these socio-cultural factors were not quantitively analyzed in the current study and could be further investigated in following studies (Add some reference: agreement with literature, Cultural studies).

**TABLE 6:** Reasons for patients not choosing to live in nursing facilities.

Item	*N*	%
**Subjective factors**		
Disagreement of the patient’s family members.	614	36.7
Disagreement of the patient him/herself.	558	33.3
**Objective factors**		
No local nursing facilities.	108	6.4
Too long of the queuing time.	137	8.2
Insufficient service level of nursing facilities.	578	34.5
Too much expense of the nursing facilities.	584	34.9
Unable to move in due to physical condition (illness, disability, etc.).	199	11.9
Once lived in, but later decided against nursing facility.	74	4.4

Most AD patients will deteriorate to a completely dependent state along disease trajectory, placing have care burdens to their families both economically and sociologically. An international multilateral cost-of-illness (COI) studies has summarized that the socioeconomic cost of AD includes direct medical, direct non-medical and indirect costs (Callahan, 2017). Jia et al. (2018) have predicted that the annual cost of AD patients worldwide to be US $9.12 trillion in 2050. The burden enforced by AD on families is not just financial, but also affects other aspects of life. For example, the symptoms of dementia often cause physical, emotional, and mental stress (D’Onofrio et al., 2015). Many studies have explored the influence factors of caregiver burden, indicating that the burden on caregivers is higher in families with lower income and disease severity (Montgomery et al., 2018; Kawano et al., 2020). In addition, disease related burden for family caregivers of AD patients increases drastically as disease progresses, and is influenced by the caregiver’s education, and being spouse of the patient (Lou et al., 2015; Liu et al., 2017b). Most families experience mental tension due to AD and a negative psychological interactions between caregivers and patients has been reported (Andren and Elmstahl, 2008). However, there are few recent and large-scale, multicenter studies on caregiver burden of Chinese AD patients, and most studies have focused on patients receiving family care (Yu et al., 2015; Liu et al., 2017a; Zhang et al., 2018). Hence, there is a lack of research on the impact of care style on family burden inflicted by AD in Chinese population.

According to the current study, the self-care ability and the burden on family members has a significant impact on care status of AD patients. The families who choose a nursing home for their elderly who lost self-care abilities usually have suffered a higher burden before and even after the decision of institutional care is made. Underdeveloped social nursing facilities in China also bring many concerns to families of AD patients, the care of whom is different from ordinary elderlies, and providing both medically and psychologically professional care for demented patients is an imminent problem for the institutional care systems in China. On the other hand, the social insurance system also results in a higher cost of living in a nursing home for the patients’ families, as reflected in by the influence of the patient’s personal income on the choice of care type. Further, the general public, including the family members’ lack of awareness and understanding of AD may also have negatively influence the caregiving status of AD patients and social burdens for their families (Dai et al., 2015; Zeng et al., 2015). Other aspects might also affect the choice, as studies have suggested that most families willing to send their elderly to nursing homes have a higher awareness of diseases, while less-caring families have lower perception of burden and tend to choose family care (Jia et al., 2020).

The current study has its implications for the improvement of China’s social insurance system, as it reflects a lack of accessible and professional nursing assistance for patients and families impacted by AD (Samus et al., 2018). For Chinese patients with AD, there are restricted alternatives and only few choices to live their lives with financial constraints (Zeng et al., 2020). According to a study in 2015, most AD patients in China have two offspring or more (80.56%), while 19.44% of participants have only one child or no child (Jia et al., 2018). However, the consequence of the one-child policy is changing the scenario dramatically in the upcoming decades, and a foreseeable challenge to the social care system is imminent, with a simulation study in has projected the economic burden associated with AD to increase by 37-fold by 2050 compared 2011 (Keogh-Brown et al., 2016). On the other hand, a more capable social support system (aside from financial aid) needs to be established both for the patients and the family members to ease their distress (Patterson et al., 1998; Wang Z. et al., 2018). Therefore, early warns should be given to policymakers to take effective measures.

The current study has some limitations that should be noted. First, due to practical limitations, we were unable to perform multistage sampling to ensure a balanced geographical sample, and the current work was lead by local health centers which volunteered to cooperate (the top level of the hierarchical structure in China). While these centers are responsible for referred patients from community clinic and walk-in outpatient services are accessible for all citizens, they are usually located in urban areas the sampling process has a predilection for urban dwellers, who could not fully represent the AD patient population in China. Secondly, the questionnaire used here was only available to patients who were diagnosed as clinically probable AD dementia and further studies could expand their scope to possible AD, MCI and all-cause dementia. Thirdly, the severity of AD was not quantified with our study design and functional health outcomes were not evaluated due to the cross-section nature of the study. The economic burden and mental stress of the patients’ family could be investigated in more detail in retrospect, for example, the service time of a family member as care giver would an important factor. All these problems warrant further investigation in a larger and more balanced patient cohort. Nevertheless, the current study has provided a basic understanding of the caregiving status and burden on Chinese families with AD patients.

In conclusion, the economic costs of AD come from all directions. Family care is the primary method of care for AD patients in China. The method of patient care is influenced by the housing condition, patient income, and disease severity. Overall, this study reveals the present situation of AD patients and their families and provides insights to help public health policymaking.

## Data Availability Statement

The datasets presented in this article are not readily available because of institutional privacy policy. Requests to access the datasets should be directed to YS, sya@bjmu.edu.cn.

## Ethics Statement

The studies involving human participants were reviewed and approved by the Ethics Committee of Peking University First Hospital. The patients/participants provided their written informed consent to participate in this study.

## Author Contributions

YL and FL contributed to the analysis and interpretation of the data, drafting and revision of the manuscript. QX, JZ, AD, FZ, WS, LC, HW, and HX contributed to collection of data, quality control, and establishing the database. XK contributed to advertising and coordination of the study. FG, HJ, and YS contributed to the conceptualization of the study, formulation of study protocol and intellectual revision of the manuscript. All authors contributed to the article and approved the submitted version.

## Conflict of Interest

XK was employed by The People’s Daily, China. The remaining authors declare that the research was conducted in the absence of any commercial or financial relationships that could be construed as a potential conflict of interest.

## Publisher’s Note

All claims expressed in this article are solely those of the authors and do not necessarily represent those of their affiliated organizations, or those of the publisher, the editors and the reviewers. Any product that may be evaluated in this article, or claim that may be made by its manufacturer, is not guaranteed or endorsed by the publisher.

## References

[B1] Afram B. Stephan A. Verbeek H. Bleijlevens M. H. Suhonen R. Sutcliffe C. (2014). Reasons for institutionalization of people with dementia: informal caregiver reports from 8 European countries. *J. Am. Med. Dir. Assoc.* 15 108–116. 10.1016/j.jamda.2013.09.012 24238605

[B2] Alzheimer’s Association (2018). 2018 Alzheimer’s disease facts and figures. *Alzheimers Dement.* 14 367–429. 10.21926/obm.geriatr.1904079 31737867 PMC6857807

[B3] Andren S. Elmstahl S. (2008). The relationship between caregiver burden, caregivers’ perceived health and their sense of coherence in caring for elders with dementia. *J. Clin. Nurs.* 17 790–799. 10.1111/j.1365-2702.2007.02066.x 18279282

[B4] Atri A. (2019). The Alzheimer’s Disease Clinical Spectrum: diagnosis and Management. *Med. Clin. North. Am.* 103 263–293. 10.1016/j.mcna.2018.10.009 30704681

[B5] Bokberg C. Ahlstrom G. Leino-Kilpi H. Soto-Martin M. E. Cabrera E. Verbeek H. (2015). Care and Service at Home for Persons With Dementia in Europe. *J. Nurs. Scholarsh.* 47 407–416. 10.1111/jnu.12158 26255994

[B6] Callahan C. M. (2017). Alzheimer’s Disease: individuals, Dyads, Communities, and Costs. *J. Am. Geriatr. Soc.* 65 892–895. 10.1111/jgs.14808 28474413

[B7] Chou K. R. Jiann-Chyun L. Chu H. (2002). The reliability and validity of the Chinese version of the caregiver burden inventory. *Nurs. Res.* 51 324–331. 10.1097/00006199-200209000-00009 12352781

[B8] Dai B. Mao Z. Wu B. Mei Y. J. Levkoff S. Wang H. (2015). Family Caregiver’s Perception of Alzheimer’s disease and caregiving in Chinese culture. *Soc. Work Public Health* 30 185–196. 10.1080/19371918.2014.969858 25602761

[B9] D’Onofrio G. Sancarlo D. Addante F. Ciccone F. Cascavilla L. Paris F. (2015). Caregiver burden characterization in patients with Alzheimer’s disease or vascular dementia. *Int. J. Geriatr. Psychiatry* 30 891–899. 10.1002/gps.4232 25475248

[B10] Genet N. Boerma W. G. Kringos D. S. Bouman A. Francke A. L. Fagerstrom C. (2011). Home care in Europe: a systematic literature review. *BMC Health Serv. Res.* 11:207. 10.1186/1472-6963-11-207 21878111 PMC3170599

[B11] Jia J. Wang F. Wei C. Zhou A. Jia X. Li F. (2014). The prevalence of dementia in urban and rural areas of China. *Alzheimers. Dement.* 10 1–9. 10.1016/j.jalz.2013.01.012 23871765

[B12] Jia J. Wei C. Chen S. Li F. Tang Y. Qin W. (2018). The cost of Alzheimer’s disease in China and re-estimation of costs worldwide. *Alzheimers. Dement.* 14 483–491. 10.1016/j.jalz.2017.12.006 29433981

[B13] Jia L. Quan M. Fu Y. Zhao T. Li Y. Wei C. (2020). Dementia in China: epidemiology, clinical management, and research advances. *Lancet Neurol.* 19 81–92. 10.1016/S1474-4422(19)30290-X 31494009

[B14] Kawano Y. Terada S. Takenoshita S. Hayashi S. Oshima Y. Miki T. (2020). Patient affect and caregiver burden in dementia. *Psychogeriatrics* 20 189–195. 10.1111/psyg.12487 31698515

[B15] Keogh-Brown M. R. Jensen H. T. Arrighi H. M. Smith R. D. (2016). The Impact of Alzheimer’s Disease on the Chinese Economy. *EBioMedicine* 4 184–190. 10.1016/j.ebiom.2015.12.019 26981556 PMC4776062

[B16] Lee T. W. Yim E. S. Choi H. S. Chung J. (2019). Day care vs home care: effects on functional health outcomes among long-term care beneficiaries with dementia in Korea. *Int. J. Geriatr. Psychiatry* 34 97–105. 10.1002/gps.4992 30246886

[B17] Liu S. Jin Y. Shi Z. Huo Y. R. Guan Y. Liu M. (2017a). The effects of behavioral and psychological symptoms on caregiver burden in frontotemporal dementia, Lewy body dementia, and Alzheimer’s disease: clinical experience in China. *Aging Ment. Health* 21 651–657. 10.1080/13607863.2016.1146871 26882509

[B18] Liu S. Li C. Shi Z. Wang X. Zhou Y. Liu S. (2017b). Caregiver burden and prevalence of depression, anxiety and sleep disturbances in Alzheimer’s disease caregivers in China. *J. Clin. Nurs.* 26 1291–1300. 10.1111/jocn.13601 27681477

[B19] Lou Q. Liu S. Huo Y. R. Liu M. Liu S. Ji Y. (2015). Comprehensive analysis of patient and caregiver predictors for caregiver burden, anxiety and depression in Alzheimer’s disease. *J. Clin. Nurs.* 24 2668–2678. 10.1111/jocn.12870 26108739

[B20] Luppa M. Luck T. Weyerer S. Konig H. H. Brahler E. Riedel-Heller S. G. (2010). Prediction of institutionalization in the elderly. A systematic review. *Age Ageing* 39 31–38. 10.1093/ageing/afp202 19934075

[B21] McKhann G. M. Knopman D. S. Chertkow H. Hyman B. T. Jack C. R. Jr. Kawas C. H. (2011). The diagnosis of dementia due to Alzheimer’s disease: recommendations from the National Institute on Aging-Alzheimer’s Association workgroups on diagnostic guidelines for Alzheimer’s disease. *Alzheimers. Dement.* 7 263–269. 10.1016/j.jalz.2011.03.005 21514250 PMC3312024

[B22] Montgomery W. Goren A. Kahle-Wrobleski K. Nakamura T. Ueda K. (2018). Alzheimer’s disease severity and its association with patient and caregiver quality of life in Japan: results of a community-based survey. *BMC Geriatr.* 18:141. 10.1186/s12877-018-0831-2 29898679 PMC6000944

[B23] Novak M. Guest C. (1989). Application of a multidimensional caregiver burden inventory. *Gerontologist* 29 798–803. 10.1093/geront/29.6.798 2516000

[B24] Patterson T. L. Semple S. J. Shaw W. S. Yu E. He Y. Zhang M. Y. (1998). The cultural context of caregiving: a comparison of Alzheimer’s caregivers in Shanghai, China and San Diego, California. *Psychol. Med.* 28 1071–1084. 10.1017/s0033291798007053 9794014

[B25] Pei J. J. Giron M. S. Jia J. Wang H. X. (2014). Dementia studies in Chinese populations. *Neurosci. Bull.* 30 207–216. 10.1007/s12264-013-1420-1 24627330 PMC5562664

[B26] Prince M. Dementia Research Group (2004). Care arrangements for people with dementia in developing countries. *Int. J. Geriatr. Psychiatry* 19 170–177. 10.1002/gps.1046 14758582

[B27] Samus Q. M. Black B. S. Bovenkamp D. Buckley M. Callahan C. Davis K. (2018). Home is where the future is: the BrightFocus Foundation consensus panel on dementia care. *Alzheimers. Dement.* 14 104–114. 10.1016/j.jalz.2017.10.006 29161539 PMC5870894

[B28] Wang Y. Xiao L. D. Luo Y. Xiao S. Y. Whitehead C. Davies O. (2018). Community health professionals’ dementia knowledge, attitudes and care approach: a cross-sectional survey in Changsha, China. *BMC Geriatr.* 18:122. 10.1186/s12877-018-0821-4 29801476 PMC5970511

[B29] Wang Z. Ma C. Han H. He R. Zhou L. Liang R. (2018). Caregiver burden in Alzheimer’s disease: moderation effects of social support and mediation effects of positive aspects of caregiving. *Int. J. Geriatr. Psychiatry* Epub online ahead of print. 10.1002/gps.4910 29856091

[B30] Yu H. Wang X. He R. Liang R. Zhou L. (2015). Measuring the Caregiver Burden of Caring for Community-Residing People with Alzheimer’s Disease. *PLoS One* 10:e0132168. 10.1371/journal.pone.0132168 26154626 PMC4496054

[B31] Zeng F. Xie W. T. Wang Y. J. Luo H. B. Shi X. Q. Zou H. Q. (2015). General public perceptions and attitudes toward Alzheimer’s disease from five cities in China. *J. Alzheimers. Dis.* 43 511–518. 10.3233/JAD-141371 25096611

[B32] Zeng Q. Wang Q. Zhang L. Xu X. (2020). Comparison of the Measurement of Long-Term Care Costs between China and Other Countries: a Systematic Review of the Last Decade. *Healthcare* 8:117. 10.3390/healthcare8020117 32365633 PMC7348717

[B33] Zhang M. Chang Y. P. Liu Y. J. Gao L. Porock D. (2018). Burden and Strain among Familial Caregivers of Patients with Dementia in China. *Issues Ment. Health Nurs.* 39 427–432. 10.1080/01612840.2017.1418034 29775139

